# Leveraging large-scale assessments for effective and equitable school practices: the case of the nordic countries

**DOI:** 10.1186/s40536-023-00172-w

**Published:** 2023-06-05

**Authors:** Nani Teig, Isa Steinmann

**Affiliations:** 1 University of Oslo, Oslo, Norway; 2 Oslo Metropolitan University, Oslo, Norway

## Abstract

One of the primary goals of educational research is to identify effective and equitable school practices that aim to promote desired educational outcomes for all students, regardless of their background. This pursuit raises the question of why certain countries or schools demonstrate more favourable outcomes than others. To shed light on this question, this special issue delves into the Nordic countries (Denmark, Finland, Iceland, Norway, and Sweden) as a showcase.

Despite their similar historical, cultural, and economic characteristics, these countries show vastly different levels of student outcomes. This special issue comprises seven studies that utilize data from the international large-scale assessments (PIRLS, TIMSS, and PISA), leveraging their international comparative design and nationally representative student samples. The present article provides an overview of the seven included studies by underlining the key themes that transcend them as well as their contributions and implications. These themes include the measurement of educational effectiveness with international large-scale assessments, the central role of teachers, and the importance of both cognitive and non-cognitive student outcomes in studying different perspectives on effective and equitable school practices.

What makes a school practice effective and equitable for all students? The answer to this question is the Holy Grail of educational research. Researchers have long sought to find out how different aspects of school systems—including teacher competence, school characteristics, and educational policies—can be optimized to provide excellent learning opportunities for diverse groups of students (e.g., Creemers & Kyriakides 2008; Hattie, 2010; Townsend, 2007). New knowledge about effective and equitable school practices is essential for evidence-based policymaking aimed at improving student outcomes and closing gaps in educational inequality.

To answer such questions, researchers have turned to national and international large-scale assessments as they offer unique research opportunities (cf. Kyriakides & Charalambous, 2014; Lietz et al., 2017; OECD, 2022). Many of these research endeavours require large datasets that encompass numerous schools and allow comparisons across countries or developments over time. Large-scale assessments, such as PIRLS (Progress in International Reading Literacy Study), TIMSS (Trends in International Mathematics and Science Study), and PISA (Programme for International Student Assessment) collect valuable repeated-measurement data using representative samples (for more information, see Mullis et al., 2017, 2020; OECD, 2019a), which offers the potential to inform educational policy and practice based on generalizable findings.

## Research focus of the special issue

This special issue presents a collection of papers that utilize the international large-scale assessments PIRLS, TIMSS, and PISA to gain a better understanding of what makes primary and secondary school practices effective and equitable. They focus on the Nordic countries (Denmark, Finland, Iceland, Norway, and Sweden) as a showcase because these countries have very similar historical, cultural, and economic characteristics, which implies that schools and teachers work under somewhat comparable conditions. Despite these commonalities, the Nordic countries show very different results in the large-scale assessments across subject areas and trends over time.

**Fig. 1:**
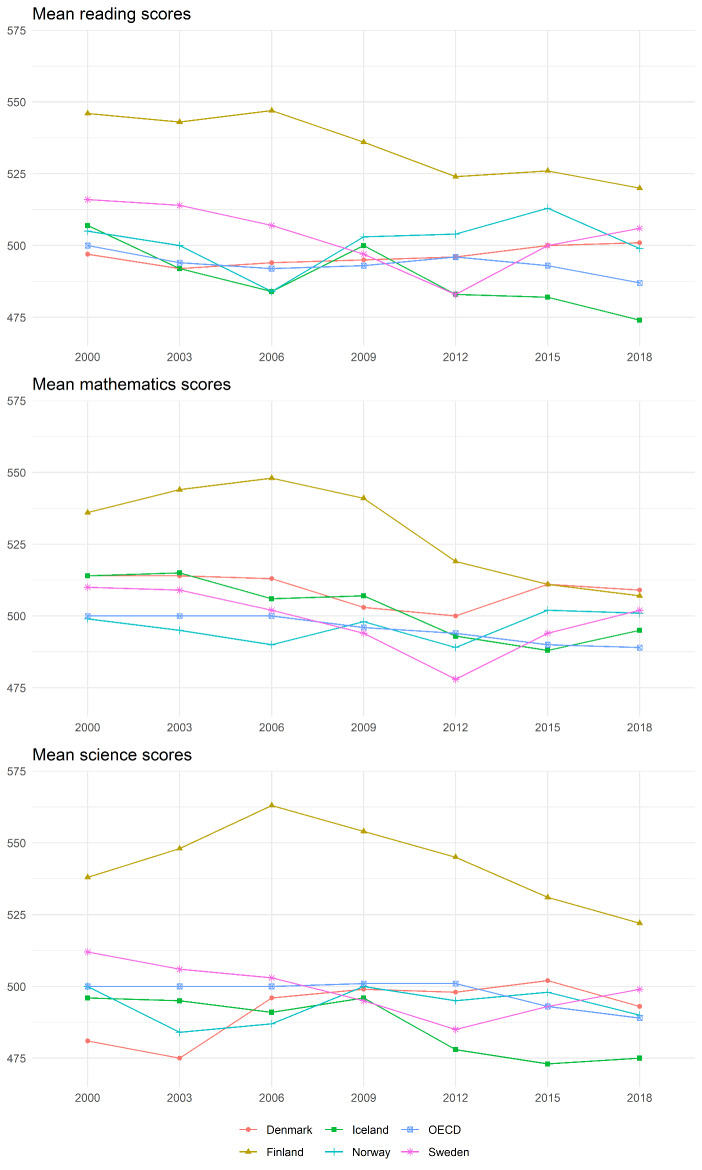
Illustration of trends in mean achievement scores in the Nordic and across OECD countries in PISA 2000‒2018. (Sources: Own illustration based on data from PISA reports (OECD, 2003, 2004, 2007, 2010, 2014, 2016, OECD, 2019b))

As Fig. 1 illustrates, the Nordic countries showed different trends in the overall scores for reading, mathematics, and science across the PISA cycles. Denmark showed relatively flat trends in reading and mathematics, but an increase in science between 2000 and 2018. Iceland’s scores, on the other hand, declined in all three domains. Finland tended to score higher than the other Nordic countries and the OECD average in all three domains, but its mean scores in reading declined between 2000 and 2018, while the mean scores in mathematics and science first increased and then decreased. Meanwhile, in Norway, the mean scores fluctuated between PISA cycles but showed overall rather stable trends. Sweden, on the other hand, showed first decreasing and then increasing trends in all three domains between 2000 and 2018.

The heterogeneity of the results across the Nordic countries (Fig. 1) indicates that the Nordic education systems and their outcomes are not as similar as one might expect, given their historical and political commonalities. These differential trends could be attributed to differences in policymaking or changes in schooling contexts, among other factors. This special issue focuses on effective and equitable school practices in the Nordic countries. The studies use PIRLS, TIMSS, or PISA data from one or more Nordic countries to investigate associations between classroom- or school-level characteristics with student-level outcomes, such as academic achievement or educational inequalities. The studies use different state-of-the-art methodological approaches to utilize the large-scale assessment data for their specific research questions. This way, the special issue combines outstanding contributions to the overarching research field of effective and equitable school practices with a focus on primary and secondary school education in the Nordic countries.

**Table 1:** Overview of articles in the special issue

Study	Data	Countries						Research focus	Research methods
		DNK	FIN	ISL	NOR	SWE	Others		
Steinmann and Olsen (2022)	− TIMSS 1995 Pop. A & B − TIMSS 2015 Pop. A & B			✓	✓	✓	− Cyprus − Greece − Scotland − Slovak Republic	Measuring the degree to which schools vary in effect of one school year on mathematics and science achievement (i.e., equality of school effectiveness)	Multilevel regression discontinuity design
Rohatgi et al. (2022)	PISA 2015	✓	✓	✓	✓	✓		Estimating association between student-perceived supportive climate in science classes and science achievement	Multilevel regression analysis with control variables
Yang Hansen et al. (2022)	− PISA 2000 − PISA 2018	✓	✓		✓	✓	− Hong Kong − Beijing, Shanghai, Jiangsu, Zhejiang	Investigating associations between school contexts and student achievement and academic self-concept in reading	Multiple-group multilevel structural equation modelling approach
Nilsen et al. (2022)	− TIMSS 2015 Pop. B − TIMSS 2019 Pop. B				✓			Studying link between school climate and student motivation with decline in student mathematics achievement	Mediation structural equation modelling with trend design
Siebecke and Jarl (2022)	− PISA 2000 − PISA 2003 − PISA 2006 − PISA 2009 − PISA 2012 − PISA 2015 − PISA 2018					✓		Identifying resilient schools in Sweden and whether material well-being at schools can compensate for socioeconomic disadvantages	The Kruskal-Wallis one-way ANOVA on ranks and pairwise comparison
Leino et al. (2022)	PIRLS 2016	✓	✓		✓	✓		Examining the associations among teacher quality and instructional quality with cognitive and affective-motivational student outcomes	Structural equation modelling
Costa and Chen (2023)	PISA 2012	✓			✓	✓		Demonstrating the potential of examining process data from large-scale assessment to gain a deeper understanding of student performance	Latent regression modelling

## Contributions and implications of the special issue studies

The seven studies included in this special issue add to the existing body of research by examining various factors that contribute to effective and equitable school practices. The implications of these findings are relevant for policymakers, stakeholders, and practitioners in education in their efforts towards improving educational outcomes and promoting equity in education. Table 1 summarizes the studies included in this special issue.

Steinmann and Olsen (2022) proposed an innovative method to measure countries’ equality of school effectiveness. They utilized a multilevel regression discontinuity method to disentangle baseline achievement levels of schools, the effect of student age, and the schools’ effects of an additional year of schooling (i.e., school effectiveness). The method was applied to a number of countries that participated in TIMSS 1995 and 2015. The unique aspect of this approach was the utilization of two adjacent grade levels (e.g., Grades 4 and 5) for the participating countries, rather than a single grade level (e.g., Grade 4). Key findings revealed that some countries, such as Norway, attained high degrees of equality of school effectiveness, even though their schools varied in baseline achievement levels. Furthermore, schools with disadvantaged student bodies had lower achievement levels than more privileged schools, but the socioeconomic composition was usually not related to the degree of school effectiveness. Thus, the study suggests that schools can achieve high effectiveness levels, even with socioeconomically disadvantaged students.

Siebecke and Jarl (2022) used PISA data from Sweden between 2000 and 2018 to examine resilient schools that demonstrate high academic outcomes despite having a student body that is predominantly from low socioeconomic backgrounds. This study identified a substantial decrease in resilient schools, indicating a decline in educational equity in Sweden. Policymakers need to address this issue and can learn from the group of resilient schools that were able to mitigate the impact of socioeconomic disadvantages on student achievement. While this study did not find any significant differences in material well-being between resilient and non-resilient schools, there was a consistent pattern of disadvantaged schools experiencing learning challenges due to teacher shortages and insufficiently qualified teachers compared to advantaged schools. The study argues that addressing teacher shortages and ensuring a compensatory allocation of resources to disadvantaged schools can help to ensure equal access to high-quality education for all students regardless of their socioeconomic status.

Rohatgi et al. (2022) examined students’ perceptions of supportive climate — namely, teacher support, teacher fairness, perceived feedback, and disciplinary climate — and their relations to science achievement across Nordic countries using PISA 2015 data. The study found that teacher fairness and feedback had the strongest association with students’ science achievement, both at the student and school levels, even when controlling for relevant student, family, and school variables. However, a high frequency of teacher feedback was more commonly reported in low-achieving students and schools. These findings emphasize the important role of supportive teacher-student relationships in enhancing educational outcomes and highlight the need for effective teacher training and professional development to promote a supportive learning environment.

Nilsen et al.’s (2022) study aimed to identify factors contributing to the decline in the mathematics performance of students in Norway from TIMSS 2015 to 2019. This study showed that the decline in students’ perceptions of a safe school environment and their self-concept in learning mathematics was significantly related to the decline in mathematics performance. These findings have implications for policymakers, highlighting the need to address school climate issues to ensure all students have access to a safe learning environment. The study also emphasizes the importance of considering students’ attitudes and beliefs about their abilities in developing instructional strategies that can enhance student performance in mathematics.

Yang Hansen et al. (2022) addressed the role of school context factors on student reading achievement and academic self-concept outcomes. Specifically, the study focused on socioeconomic and academic school compositions. They used PISA 2000 and 2018 data from four Nordic countries and two regions in China. The findings indicate that schools became more homogeneous in terms of their socioeconomic and academic compositions in most of these countries. The authors discuss these findings in light of varying educational policies in the respective education systems. As hypothesized, the study identified a positive relation between the socioeconomic school composition and student achievement, and a negative relation between the academic school composition and students’ self-concepts. These findings contribute to understanding the Big-Fish-Little-Pond effect, a counterintuitive phenomenon in which students in high-achieving classrooms tend to have lower academic self-concepts compared to students with similar abilities who attend lower-achieving classrooms.

In their study using PIRLS 2016 data, Leino et al. (2022) identified significant but weak associations between teacher quality and instructional quality with student cognitive and affective-motivational outcomes in reading. Additionally, the strength of these associations slightly varied between the Nordic countries. This study highlights three important factors for teacher training and professional development: teacher self-efficacy, teacher support, and conducive learning atmosphere. Equipping teachers with knowledge and resources in these areas is crucial for reducing gender and socioeconomic disparities in students’ reading performance and attitudes. Policymakers and educational stakeholders should consider emphasizing specialized training in reading instruction and promoting participation in reading-related professional development to improve teaching quality and student outcomes in Nordic countries.

Costa and Chen (2023) analysed PISA 2012 data from Norway, Sweden, and Denmark to gain a deeper insight into students’ performance by examining their characteristics and behaviours when answering mathematics tasks. This study revealed that students’ performance in mathematics, their exploration behaviours during problem-solving, and response time varied across countries and were partially related to students’ gender, socioeconomic background, access to information and communications technology, and experience with pure or applied mathematics tasks. The findings of this study highlight the potential of using fine-grained process data from computer-based assessments to better understand the underlying factors contributing to the differences in students’ achievement scores. These findings could aid test designers in creating fair and equitable assessment tasks for diverse learners and inform evidence-based policymaking to develop effective instructional strategies that address differences in students’ characteristics.

## Concluding remarks

This special issue features seven articles that explore different perspectives on effective and equitable school practices using Nordic countries as examples. Steinmann and Olsen (2022) highlight that some countries, such as Norway, already exhibit a high degree of equality of school effectiveness. Some countries exhibited low levels of between-school variation in student learning gains over one school year. Moreover, while schools with disadvantaged student populations tended to have lower mean achievement levels, they do not necessarily show lower mean achievement gains between two school years. This study offers encouraging findings for advancing equity in education.

While the Nordic countries differ in mean achievement levels and trends over time (see Fig. 1), there are substantial similarities in the factors that are relevant for effective and equitable school practices. One such factor is the critical role of teachers in providing a supportive learning environment and high-quality instruction (Leino et al., 2022; Nilsen et al., 2022; Rohatgi et al., 2022; Siebecke & Jarl, 2022). Teachers and their instruction are crucial for student outcomes and have the potential to reduce gaps in educational outcomes among different groups of students. Hence, the studies support the longstanding claims that policymakers and educational stakeholders need to consider strategies to ensure that all students have equitable access to high-quality teachers. These strategies may include targeted recruitment efforts to attract teachers to disadvantaged schools, financial incentives for teachers who work in these schools, and professional development opportunities to support teachers’ ongoing growth and development.

Another recurring finding in this special issue is related to the importance of considering students’ non-cognitive outcomes, such as self-concept, motivation, and attitude, in promoting educational equity (Leino et al., 2022; Nilsen et al., 2022; Yang Hansen et al., 2022). Non-cognitive learning outcomes are crucial because students with high motivation, strong self-concept, or positive attitudes towards learning are more likely to engage in learning activities and achieve academic success (Leino et al., 2022; Nilsen et al., 2022). However, students in high-achieving schools may develop lower self-concepts as they compare themselves to high-achieving peers (Yang Hansen et al., 2022). The school environment, including students’ interactions with teachers and peers, the school culture and climate, and the quality of instruction, shapes students’ experiences. Therefore, schools must adopt policies and practices that support positive socio-emotional development to create a more effective and equitable learning environment for all students.

As in any analyses using international large-scale assessment data, the studies in this special issue also encountered a common limitation related to the cross-sectional design of the data, which hinders the tracking of changes at the student level over time and establishing cause-and-effect relationships. To address this limitation, TIMSS Longitudinal study — an extension of the traditional TIMSS 2023 assessment — offers promise by following the same cohort of students over time. This study allows to track changes at the student level and to draw more robust causal inferences about student learning. However, it should be noted that only Sweden decided to participate in TIMSS Longitudinal, which highlights the need for other Nordic countries to participate as well. Another potential avenue for causal inference from the international large-scale assessments is to extend the TIMSS international design by assessing two grades per school instead of one. As demonstrated in Steinmann and Olsen’s (2022) study, this approach can identify the (equality of) the effectiveness of schooling independent of selection and age effects. Additionally, it can be used to test whether schools with certain characteristics, such as those with specific resources or a large share of qualified teachers, attain greater added-year effects than others. This approach can be extended to international designs of large-scale assessment studies with relatively low additional costs and efforts (Angrist & Pischke, 2015; Sandsør et al., 2023; Steinmann & Olsen, 2022).

In today’s world, where the COVID-19 pandemic, war, and energy crises have exacerbated educational inequalities, understanding effective and equitable school practices is more crucial than ever. Thus, special issues like the present one offer valuable insights to policymakers and educational stakeholders seeking to address these challenges. By prioritizing effective and equitable school practices, we can ensure that all students have access to high-quality education, regardless of their background and circumstances, particularly during these challenging times.

## Data Availability

Not applicable.
